# Bridging Needs and Expectations of Individuals With Physical Disabilities and Community Services Stakeholders for the Cocreation of an Adapted Physical Activity Platform in Virtual Reality: Qualitative Study

**DOI:** 10.2196/59704

**Published:** 2025-01-17

**Authors:** Aurélien Ramos, Maxence Boisvert, Elodie Traverse, Danielle Levac, Martin Lemay, Marika Demers, Martine Bordeleau, Sarah-Maude Ruest, Roxanne Périnet-Lacroix, Krista L Best, Maxime T Robert

**Affiliations:** 1 School of Rehabilitation Sciences, Université Laval, Québec, QC, Canada; 2 Centre interdisciplinaire de recherche en réadaptation et en intégration sociale, Research Center, 525 Bd Wilfrid-Hamel, Québec, QC, G1M 2S8, Canada, 1 418 649-3735; 3 Centre hospitalier universitaire Sainte-Justine, Research Center, Montreal, QC, Canada; 4 School of Rehabilitation, Faculty of Medicine, Université de Montréal, Montréal, QC, Canada; 5 Department of Physical Activity Sciences, Université du Québec a Montreal, Montréal, QC, Canada; 6 Division of Biokinesiology and Physical Therapy, University of Southern California, Los Angeles, CA, United States; 7 Centre intégré universitaire de santé et de services sociaux de l'Estrie, Centre hospitalier universitaire de Sherbrooke, Sherbrooke, QC, Canada; 8 Faculty of Medicine and Health Sciences, Université de Sherbrooke, Sherbrooke, QC, Canada; 9 Adaptavie, Community Organization, Québec, QC, Canada

**Keywords:** virtual reality, physical activity, community organizations, accessibility, physical disability

## Abstract

**Background:**

Physical activity supports the health and well-being of individuals with physical disabilities. Despite the significance of engaging in physical activity, barriers faced by individuals with disabilities, such as limited access to adapted facilities and lack of transportation, can restrict their participation. Community organizations play a role in addressing these challenges, but virtual reality (VR) also offers a way to diversify adapted activities. In some situations, VR can help overcome the resource limitations of organizations by providing accessible, engaging, and highly personalized options for physical activity.

**Objective:**

The aim of this study was to explore the needs and expectations of individuals with physical disabilities and their interventionists for the use of a VR physical activity platform in a community organization.

**Methods:**

A descriptive qualitative study was conducted using semistructured interviews with individuals with physical disabilities and their interventionists, all associated with a nonprofit organization promoting physical activity among people with disabilities. Data were analyzed using an inductive thematic approach.

**Results:**

In total, 15 participants, including 8 people with physical disabilities and 7 interventionists, were interviewed. Through this discussion, we gained insights into the everyday challenges faced by individuals with disabilities and identified priorities for community organizations. Subsequently, we discussed key considerations for using VR, including adapting activities, the possibility of fostering a more positive perception of physical activity, and harnessing the potential of VR to improve access to physical activity. We also discussed the importance of supporting personal goals and creating inclusive experiences while recognizing challenges such as technical difficulties and connectivity issues.

**Conclusions:**

By exploring the needs and expectations regarding VR technology from individuals with physical disabilities and their interventionists, this study provided essential insights for integrating immersive and nonimmersive VR into community organizations, informing next steps for the design of adapted physical activities in VR.

## Introduction

Physical activity is a cornerstone of health and well-being, yet individuals with physical disabilities face barriers in access to physical activity opportunities compared to their nondisabled peers [1]. According to Statistics Canada, of the 6.2 million people living with at least one disability in Canada, more than half do not engage in any leisure-time physical activity, and less than 15% are considered physically active [2]. Promoting access to physical activity among individuals with physical disabilities is important to prevent comorbidities [3], reduce the risk factors associated with sedentary behaviors [4], and increase participation in sports, social, and community activities [5].

Access to physical activity opportunities poses a significant challenge for individuals with physical disabilities. These barriers are multifaceted, stemming from both personal and environmental factors that impede access to physical activity. On the environmental front, difficulties are mainly due to a lack of facilities and equipment adapted to the needs of people with physical disabilities [1,5-8], inadequate means of transport, and a lack of professionals trained to work with this population [6]. These problems are particularly pronounced in rural areas, where resources are often more limited. Personal factors among people with physical disabilities, such as fear of failure, negative self-perception, or negative perception of the practice environment [1,7,9], play an important role in access to physical activities. This gap in accessible physical activity underscores the importance of community services in supporting continued engagement and overcoming these barriers.

Community services can be defined as associations that aim to improve the well-being of a community by addressing its specific needs, often through volunteer-driven initiatives and social support programs. For the purpose of this research, community services include recreational centers with adapted social and physical activities, accessible gyms with a wide heterogenous clinical profile, sports, social clubs, as well as healthy support programs. Community services can greatly influence one’s engagement in physical activity, particularly after discharge from rehabilitation services. Community-based approaches for promoting physical activity take into account the dynamic interplay of social, organizational, cultural, socioeconomic, environmental, and policy influences on an individual’s functioning [10]. Consequently, community-based interventions, defined as interventions led by therapists to improve various health-related domains (motor, cognitive, social, etc), have emerged as essential strategies for promoting engagement in physical activity [11]. Despite evidence of their positive impact on preventing secondary conditions and on improving overall health and function in people with disabilities, developing tailored programs to meet individual needs remains challenging. Adapted and accessible physical activities are essential for all individuals, regardless of health status or ability level [8,12,13]. A greater understanding of options for accessible physical activities tailored to individuals with physical disabilities is essential.

Virtual reality (VR), defined as the use of computer-generated interactive simulations, comprises a spectrum of technologies varying in type and technical intricacies, spanning from fully immersive (head-mounted displays [HMDs]) to nonimmersive (Nintendo Wii, Nintendo Switch, PlayStation, Xbox, etc) technologies [14]. VR allows users to engage in physical activities at different levels of immersion in digital landscapes where they can participate in games and tasks that are both appealing and adaptive. The growing adoption of VR, highlighted by its global market valuation of US $59.96 billion in 2022 [15], underscores its potential in various domains, including rehabilitation. One of the primary reasons VR is considered an accessible option for physical activity is its ability to create stimulating, interactive, and multimodal environments tailored to individual needs. Advantages of both nonimmersive and immersive VR interaction include promoting motor learning, motivation, adapting activities to users’ needs, and developing a sense of confidence [13,14,16-18]. Over the past decade, numerous studies, both in children and adults with physical disabilities, have highlighted the effectiveness of immersive VR for improving locomotion [18], postural control [19], upper limb functions [20], and physiological outcomes [21]. Moreover, VR offers significant potential for improving cognitive functions [22], such as attention and perception [23]. VR is recognized for sustaining motivation during sessions, thereby enhancing engagement levels and adherence to both therapeutic exercises and physical activities [24,25]. In this way, the effectiveness of VR in enhancing the accessibility of physical activity for people with disabilities has been increasingly acknowledged [26,27].

The practical application and integration of this technology within community settings, such as the accessibility of hardware to individuals that may not be familiar with this technology, remain unexplored. Indeed, the use of these technologies in community settings requires careful consideration. The transition from laboratory use to actual practice in community settings can raise challenges such as safety, space organization, training requirements, and ease of use. The aim of this study was to identify the needs and expectations of people with physical disabilities and their interventionists for the use of a VR physical activity platform in community organizations.

## Methods

### Study Design

A descriptive qualitative study was conducted using semistructured interviews.

### Participants and Recruitment

Individuals with physical disabilities and their interventionists (students or professionals in health-related domains such as physical therapists and kinesiologists), who were associated with a respite camp managed by the nonprofit organization (Adaptavie [28]), were recruited using a convenience sampling approach. No screening process was conducted, as our goal was to capture the broad spectrum of experiences of individuals engaging with the community organization, regardless of their specific disability. To be included in this study, individuals with physical disabilities and interventionists needed to have an active involvement in the summer services for a minimum of 2 weeks and written and oral French proficiency. Participants needed to be able to follow simple instructions.

### Setting

The interviews took place in an elementary school that was being used by the community partner (Adaptavie) to offer respite camps. The overarching goal of Adaptavie, a nonprofit community-based organization, is to facilitate physical activity and encourage healthy lifestyles for people with disabilities, with the aim to improve autonomy, socialization, well-being, and global health for this community. Aligned with Adaptavie’s goals, the study was integrated into the respite camps that were offered in the summer and during weekends.

### Procedure

Participants completed a ***s***ociodemographic questionnaire including age and gender. For individuals with physical disabilities, additional questions included the medical diagnosis or type of disability, occupation, assistive technology use, and the frequency of practice of physical activity weekly. For interventionists, information about professional background and educational trajectory, years of experience working with individuals with disabilities, and years of experience within Adaptavie was collected.

The research team developed 2 interview guides—one for individuals with physical disabilities and one for interventionists—through an iterative process (repeatedly refining with input from all stakeholders). These guides were designed to explore and understand the challenges of everyday life and access to physical activities for individuals with physical disabilities, as well as the interventionists’ perceptions of various types of adapted physical activities at Adaptavie. The guides also examined previous experiences with VR and perspectives on using VR to promote physical activity, considering participants’ viewpoints, expectations, needs, and preferences. Each technical term was accompanied by its definition to ensure clarity for all participants. The questions were then validated by 3 pilot participants (an occupational therapist, a kinesiologist, and an individual with a physical disability). This led to a content validation of both interview guides [29]. The interviews started with introductions and a presentation of the study objectives, followed by a period of discussion. The discussion was structured around 20 open-ended questions covering the following topics: (1) participant’s daily activities, (2) interventionist’s perception of community services, (3) prior VR experiences, and (4) perspectives on the use of VR during community services. Themes 1 and 2 allow us to understand the realities of individuals with physical disabilities and the workings of the community organization in order to understand if VR could address their specific needs. Theme 3 provides a nuanced perspective, enabling us to refine our understanding of the participants’ and interventionists’ views based on their previous experiences with VR, as well as their reactions to and feelings about these technologies. A PowerPoint presentation was used to illustrate the definition and types of VR (presenting various immersive and nonimmersive VR devices such as the Wii, the Kinect, VR headsets, etc).

The semistructured interviews were conducted in person during the summer respite services by MB and ET, both trained in qualitative data collection and interviewing skills. The data collection took place over 1 week, with interviews video recorded and averaging 50 minutes in duration.

### Data Analysis

Descriptive statistics were calculated for the sociodemographic data. Video files were converted to audio files and transcribed by MB and ET or through TranscribeMe [30] (a web-based transcription service). All transcripts were verified by MB to ensure accuracy. A thematic inductive approach was used to analyze the interviews [31]. The codebook was initially written by MB and ET following several verbatim readings. Subsequently, the codebook underwent continuous iterative updates throughout the analysis, adapting to emerging information from the interviews. Line-by-line coding of the interviews was conducted using NVivo software (version 1.7.1; Lumivero). To ensure coding consensus and ensure rigor and quality of the data, 20% of the interviews were independently coded by 2 research team members, MB and ET. The remaining interviews were independently coded by MB. The final versions were then reviewed and approved by the research team. Following the coding process, unique categories and themes were discerned, drawing upon the extensive expertise of the research team in the use of VR in individuals with physical disabilities and qualitative studies conducted within community organizations. The identified themes were then presented to the research team for thorough discussion and interpretation. In addition, to ensure all discussed topics were included, we reviewed the themes with 2 interventionists. Given that the interviews were conducted in French, the quotes presented in this article have been translated into English and verified by bilingual team members. To enhance the rigor of this study, we employed the Standards for Reporting Qualitative Research [32].

### Ethical Considerations

Ethical approval for this study was obtained from the CIUSSS Capitale-Nationale research ethics committee (2021-2064, 2023-2569). All participants provided informed consent and had the right to decline answering our questions or to stop the interview at any time if they wished. All collected data were anonymized and stored on the university’s server, with access restricted to the researchers involved in the project to ensure data confidentiality. A financial compensation of CAD $50 (US $35.30) was provided to all participants to cover their travel expenses.

## Results

### User Statistics

A total of 15 participants, including 8 individuals with physical disabilities aged between 18 and 28 (mean 24, SD 4.28) years and 7 interventionists aged between 19 and 31 (mean 24.87, SD 4.97) years, were interviewed. Tables1  2 present the characteristics of the individuals with physical disabilities and those of the interventionists.

**Table 1.** Sociodemographic information of participants with physical disabilities (n=7).

Characteristic	Values
Age (years), mean (SD)	24 (4.28)
Gender (male), n (%)	6 (85.71)
Medical diagnosis, n (%)	
Cerebral palsy	4 (57.14)
Duchenne muscular dystrophy	1 (14.29)
Congenital muscular dystrophy	1 (14.29)
Stroke aftereffects	1 (14.29)
Occupation, n (%)	
Student	3 (42.86)
Worker	1 (14.29)
Worker and student	2 (28.57)
N/A^a^	1 (14.29)
Technical aids, n (%)	
Motorized wheelchair	4 (57.14)
Walking cane	1 (14.29)
Wheelchair	1 (14.29)
N/A	1 (14.29)
Number of physical activities per week, n (%)	
4 times a week	2 (28.57)
2 times a week	1 (14.29)
0 times a week	4 (57.14)

a N/A: not applicable.

**Table 2.** Sociodemographic information of interventionists working in community organization (n=8).

Characteristic	Values
Age (years), mean (SD)	24.87 (4.97)
Gender, n (%)	
Female	6 (75)
Male	1 (12.5)
N/A^a^	1 (12.5)
Level of education, n (%)	
Bachelor’s degree in a health-related field	5 (62.5)
College diploma in a health-related field	2 (25)
Others	1 (12.5)
Role in the association, n (%)	
Coordinator	1 (12.5)
Interventionist	3 (37.5)
Kinesiologist	1 (12.5)
Social interventionist consultor	1 (12.5)
Special education instructor	1 (12.5)
Team leader	1 (12.5)
Years of experience within the organization, n (%)	
10 years	1 (12.5)
2 years	2 (25)
1 year	5 (62.5)

a N/A: not applicable.

### Interviews

The qualitative analysis revealed 4 themes reflecting important elements to consider when implementing VR-based physical activities in community settings: (1) daily activities of individuals with physical disabilities, (2) interventionists’ perception of community services, (3) prior VR experiences, and (4) perspectives on the use of VR during community services.

**Figure 1.**
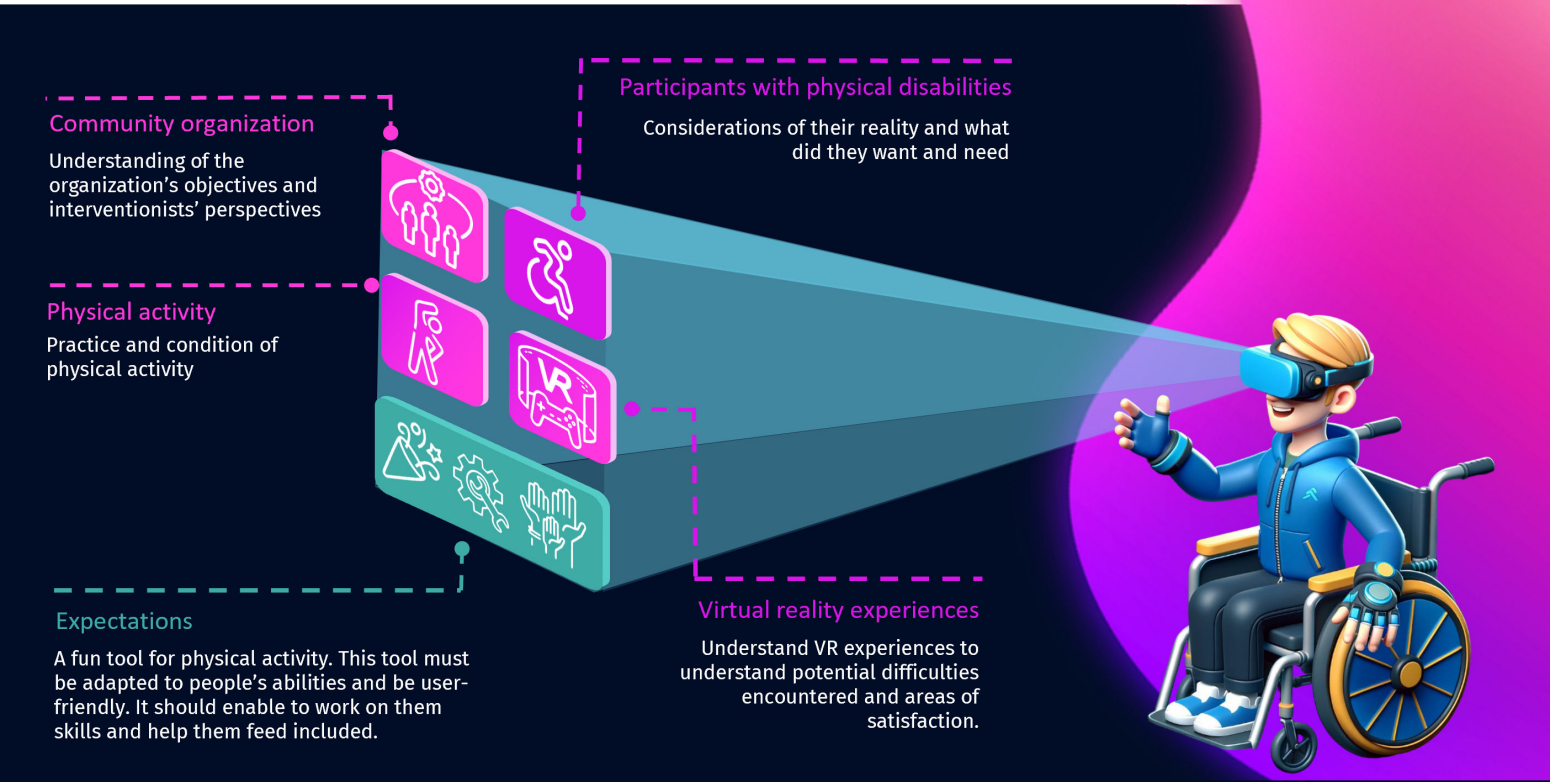
Considerations for implementing virtual reality within community organizations.

The first theme related exclusively to individuals with physical disabilities, while the second theme was relevant only to interventionists. The first 2 themes provided a better understanding of the lifestyles of the participants with physical disabilities, as well as the interventionist’s perceptions of the community services provided by the nonprofit organization. Themes 3 and 4 were applicable to interventionists and individuals with physical disabilities. Theme 3 delved into participants’ prior knowledge and experiences with VR, offering insights into their level of comfort and understanding of the technology. Theme 4, on the other hand, outlined essential considerations that must be addressed before establishing a VR platform within community services. These results are summarized in Figure 1.

### Theme 1: Daily Activities of Individuals With Physical Disabilities

All participants reported that their free time was primarily spent on passive activities, including watching television, browsing social networks, or reading. Activities of daily living reported by all participants were tasks that required the use of their upper limbs, such as cooking, dressing, reaching and grasping objects, or brushing teeth. However, 3 participants mentioned engaging in different active physical activities weekly, with neighborhood walks being the most common among all other reported activities. These physical activities were mostly conducted in services provided by a local nonprofit community organization throughout the year. Participants also expressed interest in participating in 18 physical activities, including soccer, boccia, and swimming, while completing the sociodemographic questionnaire.

Participants expressed that their motor limitations were the primary limiting factor in carrying out activities of daily living:

*Yes, there are quite a few activities that I find difficult to do. Because of my condition....When things are too heavy, or I have to throw something, or hold them in my hands….*

The participants also highlighted the lack of adaptation in their environment:

*For sure, cooking is a bit harder because my kitchen isn’t adapted at all*.

As for the practice of physical activity, several obstacles were highlighted by all participants. Most participants reported the challenge of accessing the adapted infrastructure, such as specialized gyms, which presents a significant challenge for individuals with disabilities. For example, one participant expressed frustrations over the lack of availability and accessible suitable facilities for daily practice:

*I’d like to be able to exercise at home, but space is really limited and it’s hard to get into a gym. This lack of space is a particular problem when it comes to playing games like boccia. So, I’d like to be able to practice this activity virtually*.

Some participants reported a decrease in rehabilitation services once they reached adulthood, which in turn limited their accessibility of adapted services:

*I always had followed up when I was a child and at a certain point nothing happened, it was as if by magic I was cured*.

The lack of adapted equipment was also expressed. Moreover, all participants emphasized feeling neglected in access to rehabilitation services, mainly because they perceived individuals with temporary injuries are given priority to maximize recovery:

*I find that physiotherapy prioritizes those who have accidents, because we can almost bring the person back to independence. But for us who have always had a disability....It’s as if we’ve been left on the side*.

Despite the availability of adapted physical activity programs, the public’s lack of awareness of these programs restricts opportunities for participation.

*Not many disabled people know about all the programs available to them*.

### Theme 2: Interventionist’s Perception of Community Services

The interventionists highlighted the diverse yet essential goals of the community service organizations. While these objectives vary, they all revolve around promoting the well-being of individuals. Most interventionists emphasized the significance of social integration within the community services to encourage social interactions and improve the social skills of the participants.

*Above all, we aim to promote social interaction…*

*It’s a real opportunity for our members to be in a group and work on their social skills*.

Another objective reported by most interventionists of the community services is the regular practice of (adapted) physical activity. The definition of physical activity varied significantly among interventionists, ranging from simple movement to a more detailed description of adapted physical activity. However, they all emphasized the idea of fun and entertainment in their respective descriptions of physical activity. The participant’s level of physical activity also differed from one interventionist to another. For example, while a few noted a moderate level, most interventionists reported that most participants had a low level of physical activity. Importantly, the perceived level of physical activity varied significantly among participants.

Some interventionists perceived community services as a means to promote healthy lifestyles for individuals with physical disabilities, emphasizing their role in enhancing their autonomy. Interventionists also noted that regardless of the primary objectives of community services, these programs offer rare opportunities for individuals with physical disabilities to be engaged in a wide variety of adapted activities. They also highlighted that community services also act as a respite service for participants’ families.

### Theme 3: Prior VR Experiences

Both individuals with physical disabilities and interventionists reported various levels of familiarity with the general use of VR, encompassing both immersive and nonimmersive experiences. Specifically, their familiarity with nonimmersive VR experiences ranged from 0 to over 12 years.

The systems used to experience VR, primarily for entertainment and recreation, exhibited significant diversity. Half of the individuals with physical disabilities and interventionists had experienced immersive VR devices such as the Oculus. While just under half of the interviewees mentioned experiencing immersive VR through a headset, the majority had engaged with nonimmersive VR via various video game consoles, with the Wii being notably prevalent.

Nevertheless, few individuals with physical disabilities who had prior experience of immersive VR reported side effects during prolonged use, such as dizziness and feelings of disorientation, particularly when engaging in simulations of amusement park rides:

*In fact, it was a roller coaster simulation, and you had to be seated, because the environment moves. So, the body doesn’t necessarily understand everything that’s going to happen....It’s probably mainly in the duration of exposure that, at some point, things can happen. But for me, it was like that, at the beginning, it was fine, but at some point, you go: Ouh*!

Regarding the installation of VR systems, the majority of interventionists expressed ease in using these systems:

*It’s quite simple, I think. I’m definitely not the most tech-savvy person, but despite that, I still think it’s fairly simple*.

The sentiment was echoed by other interventionists, emphasizing the increasing simplicity of technology overall:

*I’m still amazed every day at how complex it used to be, and now it’s become so accessible*.

Both individuals with physical disabilities and interventionists also reported factors influencing the ease of using VR systems, including the importance of concise and precise instructions as well as a period of familiarization. Several interventionists reported the simplicity of VR games, particularly the limited number of buttons, which is crucial in reducing complexity:

*There are things that are really simple, with not many buttons, and then there are games where you can get lost in 50 million buttons to use with lots of different levels, and all that*.

Some interventionists acknowledged that the complexity of using VR systems may vary depending on the participant’s clinical characteristics:

*And I’m still pretty sure that if there are people with physical disabilities, but who are used to playing with these kinds of consoles, they’ll be able to adapt. But maybe for people who play a little less, it can be complicated*.

Most individuals with physical disabilities reported the need for assistance when putting on a VR headset. The difficulty in using the systems varied based on specific games, equipment, and the clinical profile. In fact, many participants reported facing challenges with the controllers:

*I find the controller is not designed for people like us who have a bit of difficulty*.

Despite these technical difficulties, some participants found adaptive solutions:

*But instead of holding the controller upright, I hold it horizontally*.

### Theme 4: Perspectives on the Use of VR During Community Services

#### Advantages of Using VR Within Community Settings

Interventionists noted that VR provides numerous possibilities to improve access to physical activities and rehabilitation.

*I really think it would be great in the sense that it would open up a lot of possibilities that we don’t necessarily have right now. It’s like having access to more material. Especially in camps for people with physical disabilities, accessibility isn’t always easy*.

Interventionists highlighted the potential to assist individuals in reaching personal objectives, such as improving overall health, enhancing cognitive skills, and maximizing autonomy. Specifically, interventionists pointed out a significant benefit of VR, which involves incorporating movements needed for daily activities into a diverse array of engaging VR experiences.

*Some* [individuals with physical disabilities] *find it difficult to cook, to chop vegetables, because they have lost dexterity or other skills. So, I think that by doing it in the form of a virtual game, they could develop these abilities, but gradually, and by doing it virtually, they can’t really get hurt. It would allow them to practice, and maybe by doing it virtually, they would improve, and they could transfer it back into their daily life.*

Participants expressed a shared perspective, highlighting that VR could allow regular practice of specific movement skills essential for daily activities, all while avoiding the perception of engaging in a formal exercise:

*to help me be more mobile and less, to have less trouble picking things up*… *without it seeming like an exercise.*

#### Facilitator

Interventionists emphasized that a crucial factor for the success of VR lies in creating experiences that inspire movement and designing games that are tailored to accommodate a range of user abilities:

*VR needs to be tailored to their level, with various levels, different ways of doing things, and different settings....I just see it as a wonderful opportunity to do things beyond what they have the chance to do here*.

Participants, echoing this need, requested a variety of gameplay options (eg, 1- or 2-handed gameplay, ergonomic controller, etc) that cater to a range of physical capabilities:

*I expect it to be accessible...that I feel included.*

The VR platform should feature an intuitive and user-friendly interface.

*It would be great if it were easy to use, if it worked. That there aren’t too many unforeseen events. Of course, you can’t control it, but to say to everyone, ‘We’re going to do this,’ and then it doesn’t work. It’s kind of disappointing...I’m thinking mainly of testing. Not installing someone, and then realizing that they can’t do it. It’s always a bit disappointing.*

Creating a positive experience is key, as another interventionist noted:

*I think one of the most important objectives would be to reconcile the relation they have with physical activity, because some of them associate it with their rehabilitation, or with things that aren’t necessarily pleasant. Really make the experience positive, then, if need be, reconcile the relationship with physical activity.*

Additionally, the importance of having a dedicated support contact for assistance was highlighted. This need is complementary to the request for comprehensive training materials, such as detailed user guides and instructional videos, to effectively familiarize both interventionists and participants with the platform’s functionalities. The interventionists also emphasized the importance of a safe, practical, and user-friendly interface. They noted the need for thorough supervision of participants during equipment setup and gameplay, enabling them to observe and, if necessary, intervene.

Social interaction within the games is highlighted for fostering a sense of community, friendship, and enjoyment. Both participants and interventionists recognized the value in scenarios where players can interact, compete, or cooperate within various virtual environments.

Participants and interventionists also showed a keen interest in diverse virtual settings, covering a spectrum from modified popular commercial games to those specifically designed for physical activity. Desirable features include customizable sound settings, motivational reward systems, the ability to create and save user profiles, and improved movement sensitivity in games that cater to individuals with severe limitations.

#### Barriers and Fears

The main obstacle identified by interventionists is the heterogeneity of participants’ limitations.

*One barrier might be their limitations. It’s hard to predict for each limitation, what you can do.*

Lack of financial and human resources also emerged as major obstacles for interventionists.

*Ensuring we have the right resources for implementation is key. This means having ample financial backing for a diverse range of games. Plus, we can’t overlook the importance of human resources, especially the number of interventionists involved.*

Technological issues, such as malfunctioning equipment and poor internet connectivity, were also a concern.

#### Expected Benefits

The expected benefits of using VR are numerous, ranging from encouraging people to move while having fun, to providing access to new activities. According to an interventionist, it can enable them to move and gain confidence in their abilities:

*allow them to discover new possibilities that they might not have thought they could do....Doing things that they wouldn’t necessarily do in their everyday life, and it could give them confidence in their abilities.*

The benefits for participants vary widely. Some participants wish to engage in activities that they cannot do in the real world:

*Experiences that I can’t do because of my disability, but to be able to do them, thanks to this, might be nice.*

Others really want to maintain their skills:

*I know that playing a game won’t help me regain my mobility, but at least I’ll be able to keep it for longer.*

Some hope to become more equal with able-bodied individuals:

*Maybe become a little more equal with everyone else. In a virtual world, we can become equal again.*

## Discussion

### Principal Results

This study addressed the needs and expectations of using VR within community settings, as perceived by individuals with physical disabilities and interventionists. Our study revealed 4 essential themes for the implementation of VR within community settings, including participants’ daily activities, stakeholders’ perceptions of community services, previous VR experiences, and perspectives on the use of VR within these services.

Both individuals with physical disabilities and interventionists were enthusiastic about the possibility of having personalized VR-based interventions. In other words, the adaptability and accessibility of VR platforms were identified as crucial factors for tailoring interventions to the specific needs of individuals with physical disabilities or the organization objective. Such adaptability allows for the creation of unique experiences that are tailored to the individual’s capabilities and preferences. Ultimately, there is a strong belief in the potential of VR to improve access to physical activity, promote social integration, and foster autonomy among people with disabilities.

One of the main challenges underscored by both individuals with physical disabilities and interventionists involves maintaining the gains obtained through rehabilitation while aligning with the individual’s objectives. VR technologies offer a promising solution to engage in physical activity, serving as either a supplementary tool to rehabilitation or as a post-rehabilitation intervention [26]. One participant expressed concern about immersive VR, since it can isolate the user. This concern was of importance for this participant, as their personal objective was to alleviate loneliness and ultimately enhance social participation. Although adapting VR games to facilitate social engagement presents challenges, a recent study indicates that immersive VR can foster connections within a virtual environment, enabling individuals to support each other’s progress and thereby enriching the social aspect of rehabilitation [33].

Both individuals with physical disabilities and interventionists emphasized the importance of tailoring VR games to the clinical profiles and individuals’ goals and abilities to maximize their benefits. One approach to achieve this is by integrating motor learning principles, which involve manipulating factors such as task difficulty, repetition, motivation, and engagement [34]. Motor learning principles encompass a set of processes grounded in neuroplasticity principles associated with practice or experience, leading to relatively permanent changes in motor skills. While the primary objective of community services may not always focus on improving motor functions, our results highlighted the demand for adapted games and the aspiration to foster various skills among individuals with physical disabilities. Consequently, the development of fully customizable games could meet the expectations of both participants and interventionists by allowing all necessary elements to be adapted, added, or removed to enhance the user experience.

Crafting customizable games presents a unique blend of challenges and innovative solutions, aimed at bridging the gap between entertainment and the rehabilitative process. At the core of this initiative is the necessity to design VR games and applications that suit a diverse spectrum of interests and abilities, all the while prioritizing rehabilitation objectives. Based on the findings of this study, the goal is to design experiences that allow participants to engage in the development of various skills that resemble leisure activities more so than therapeutic interventions. However, this ambition encounters several obstacles, notably in usability and the transition from development to user experience, a transition that is often expensive and time-consuming, limiting the extent of testing and refinement needed to ensure the applications are user-friendly. This issue is particularly pronounced in rehabilitation-focused VR applications, as they cater to a more specialized audience compared to mainstream gaming. To overcome these obstacles, a structured approach to VR content development is essential, one that balances functionality with affordability. Tools like StellarX [35], which advocate for a “drag and drop” method to create VR environments without the need for coding, represent promising steps towards more accessible VR content creation.

It is important to ascertain the suitability of different game types in addressing participants’ expectations. Immersive VR has been shown to enhance the sense of presence, making virtual experiences more engaging and appealing. This is supported by advancements in VR technology, which continue to become more sophisticated and financially accessible. Nonetheless, challenges such as cybersickness with symptoms like nausea and dizziness affect many users, especially during high-motion experiences. Cybersickness is notably more prevalent in immersive VR than nonimmersive VR, with around 65.2% of users affected and 23.9% experiencing severe discomfort [36]. So this is important to think about strategy to decrease this rate as “rotation snapping and translation snapping” [37] or “foveated blur” [38]. In addition, the design of HMDs requires careful consideration to ensure positive user experiences. Studies have highlighted the advantages of HMD-VR, including elevated immersion and emotional responses, which can significantly increase satisfaction and creative freedom in gameplay [39-41].

Implementing VR in community organizations presents significant challenges, especially regarding the financial and human resources required for effective deployment. As noted by participants, it is crucial to adopt affordable technologies tailored to community organizations, ideally standalone systems that do not require additional computers. This approach aims to streamline the deployment process and ensure accessibility for community organizations with limited resources.

Finally, to effectively use VR technologies for adapted physical activity, it is crucial to understand how the technological capabilities of VR align with the specific needs of both practitioners and interventionists. This customization process involves ensuring the VR equipment is comfortable to use, providing clear and concise instructions, and offering prompt assistance to resolve any issues encountered during usage. The goal is to create an environment where professionals and individuals with physical disabilities have the skills and knowledge to use VR for rehabilitation and leisure activities. The implementation of knowledge dissemination tools, such as introductory coaching sessions, user manuals, and instructional videos, can play a significant role in achieving this goal [42].

### Limitations

One of the primary limitations of this study is the sample size. Despite our efforts to encompass a wide range of participants, the relatively small number of people involved may not fully represent the diverse populations served by community services. Another limitation of this study is the diversity of backgrounds, expertise, and varying levels of experience in VR of the interventionists who participated in this study. The heterogeneity of their academic and professional backgrounds and their varying degrees of familiarity with VR could influence their understanding of the implementation of an adapted physical activity program for people with physical disabilities.

In addition, it is important to note that while this study focused on specific types of community services, its findings may not be universally applicable across all community organizations. The diversity in operational structures, target populations, and services among community centers suggests that the considerations about implementing these kinds of interventions might vary. The findings of this study apply primarily to individuals with similar disabilities and may not extend to populations with varying medical diagnoses or a broader range of disabilities.

### Conclusions

The integration of VR to provide accessible physical activity opportunities in community services offers promising opportunities but also presents significant challenges. Tailoring VR experiences to individual needs, addressing the barriers to physical activity participation, and ensuring continuity of care from childhood to adulthood are critical areas that need attention. Community organizations play a vital role in facilitating physical activity and social integration for individuals with disabilities. Future research should focus on developing standardized, accessible, and engaging VR interventions that can be easily implemented in community settings, with appropriate resources and training for interventionists. Such an approach could provide an alternative form of physical activity designed to improve the quality of life and independence of individuals with disabilities.

## Abbreviations

HMD: head-mounted display
VR: virtual reality
